# Oscillation Phase Locking and Late ERP Components of Intracranial Hippocampal Recordings Correlate to Patient Performance in a Working Memory Task

**DOI:** 10.3389/fnhum.2016.00287

**Published:** 2016-06-16

**Authors:** Jonathan K. Kleen, Markus E. Testorf, David W. Roberts, Rod C. Scott, Barbara J. Jobst, Gregory L. Holmes, Pierre-Pascal Lenck-Santini

**Affiliations:** ^1^Department of Neurology, University of California, San FranciscoSan Francisco, CA, USA; ^2^Department of Neurology, Geisel School of Medicine at DartmouthHanover, NH, USA; ^3^Department of Surgery, Geisel School of Medicine at DartmouthHanover, NH, USA; ^4^Department of Neurological Sciences, University of Vermont College of MedicineBurlington, VT, USA; ^5^Department of Neurology, University College London, Institute of Child HealthLondon, UK; ^6^INMED Institut National de la Santé et de la Recherche Médicale U901Marseille, France; ^7^Aix Marseille Université, INMEDMarseille, France

**Keywords:** phase locking, working memory, delta, theta, alpha

## Abstract

In working memory tasks, stimulus presentation induces a resetting of intracranial temporal lobe oscillations in multiple frequency bands. To further understand the functional relevance of this phenomenon, we investigated whether working memory performance depends on the phase precision of ongoing oscillations in the hippocampus. We recorded intra-hippocampal local field potentials in individuals performing a working memory task. Two types of trials were administered. For high memory trials presentation of a list of four letters (“List”) was followed by a single letter memory probe (“Test”). Low memory load trials, consisting of four identical letters (AAAA) followed by a probe with the same letter (A), were interspersed. Significant phase locking of ongoing oscillations across trials, estimated by the Pairwise Phase Consistency Index (PPCI) was observed in delta (0.5–4 Hz), theta (5–7 Hz), and alpha (8–12 Hz) bands during stimulus presentation and recall but was increased in low memory load trials. Across patients however, higher delta PPCIs during recall in the left hippocampus were associated with faster reaction times. Because phase locking could also be interpreted as a consequence of a stimulus evoked potential, we performed event related potential analysis (ERP) and examined the relationship of ERP components with performance. We found that both amplitude and latency of late ERP components correlated with both reaction time and accuracy. We propose that, in the Sternberg task, phase locking of oscillations, or alternatively its ERP correlate, synchronizes networks within the hippocampus and connected structures that are involved in working memory.

## Introduction

There is increasing evidence that oscillatory activity in the hippocampus plays a major role in learning and memory. For instance, hippocampal theta rhythm in rodents (5–12 Hz) has been implicated in synaptic plasticity (Holscher et al., 1997; Hyman et al., 2003), neural coding (O'Keefe and Recce, 1993; Dragoi and Buzsaki, 2006), and memory encoding (Winson, 1978; Givens, 1996). In humans, in the context of intracranial recordings in patients with epilepsy, the role of delta (1–4 Hz) and theta (4–8 Hz) is beginning to be understood. As with rodents, the human hippocampus is involved in episodic memory and spatial navigation (Burgess et al., 2002; Ekstrom et al., 2003, 2005) but also in other aspects of memory, including working memory of non-spatial information (Rizzuto et al., 2003, 2006; Olson et al., 2006; Axmacher et al., 2007, 2009). In the Sternberg working memory task theta power is increased in temporal lobe intracranial electrodes during stimulus presentation, as opposed to inter-trial intervals (Raghavachari et al., 2001). In this task, participants are presented with a list of items to memorize and asked, after a delay, to determine whether test items were present in the list. Importantly, list and test stimulus presentation are followed by a systematic locking of the phase of this oscillation across trials (Tesche and Karhu, 2000; Rizzuto et al., 2003, 2006; Mormann et al., 2005). Finally, it has been demonstrated that the presentation of stimuli that are to be encoded and those that constitute a retrieval cue generate a resetting of theta oscillations at different phases (Rizzuto et al., 2006). This observation was used to support the hypothesis that specific memory functions, such as encoding and retrieval, are processed at different phases of theta oscillations (Hasselmo et al., 2002). Together with studies performed in rodents (Adey and Walter, 1963; Givens, 1996; Vinogradova et al., 1996; Holscher et al., 1997; Hyman et al., 2003; McCartney et al., 2004), these results support the notion that phase reset of ongoing theta oscillations ensures that information is transmitted at a phase that is optimal for memory processing.

The hypothesis that phase reset of ongoing oscillations supports a specific memory function is however challenged by the fact that this phenomenon is observed simultaneously in multiple frequency bands (Rizzuto et al., 2003; Mormann et al., 2005) and can be seen for both hits and correct rejections in a word recognition task (Mormann et al., 2005). In addition, the time range at which phase reset occurs corresponds to the time range of previously documented Event Related Potentials (ERPs) in intracranial hippocampal recordings, such as the Late Negative Component (LNC; Grunwald et al., 1995; Mormann et al., 2005) observed ~400 ms after stimulus presentation or late DC components (Axmacher et al., 2007, 2010). These late ERPs have been shown to vary in amplitude with working memory load (Axmacher et al., 2007, 2010) or cognitive processes (Mormann et al., 2005).

Whether the observed ERP is a consequence of phase reset of multiple ongoing oscillations (Klimesch et al., 2007) or, to the contrary the phase locking phenomenon emerges from processing of an evoked response is still a matter of debate.

To address the relevance of phase reset in working memory performance we analyzed intra-hippocampal local field potentials in patients with intractable temporal lobe epilepsy undergoing pre-surgical evaluation. We hypothesized that phase reset of delta and theta oscillations would vary with performance and memory load.

We report that phase locking in delta, theta, and alpha frequency bands increases after task stimulus presentation, but that phase consistency does not decrease in trials with lower memory load. However, across subjects, there was a correlation between phase locking and performance: patients with higher phase consistency over trials in left hippocampal delta during recall answered quicker and had a higher accuracy than patients with lower phase locking values (PLV). In contrast, patients with a higher theta phase consistency over trials had longer reaction times. ERP analysis revealed that the LNC latency and amplitude are correlated with both reaction time and accuracy. Whether phase reset of ongoing oscillations is responsible for the observed ERP or, to the contrary, whether ERP is responsible for the increased phase locking is still a matter of debate that remains to be clarified.

## Methods

### Intracranial EEG

Ten patients with refractory seizures undergoing evaluation for possible surgical resection of the epileptic zone participated in the study. The study was approved by the Committee for the Protection of Human Subjects at Dartmouth College. All patients provided informed consent to participate in the study. For patients with IQ below 70, consent was also given by their legal representative. Testing began 3–5 days following the implantation of electrodes when pain was significantly controlled and antiepileptic medications had been reduced or withdrawn to increase the likelihood of seizures for pre-surgical characterization. Patients did not participate in the study on days when they had a seizure within 6 h of starting the study, or if they did not feel well-enough to participate. No seizure occurred during testing sessions.

**Figure 1:**
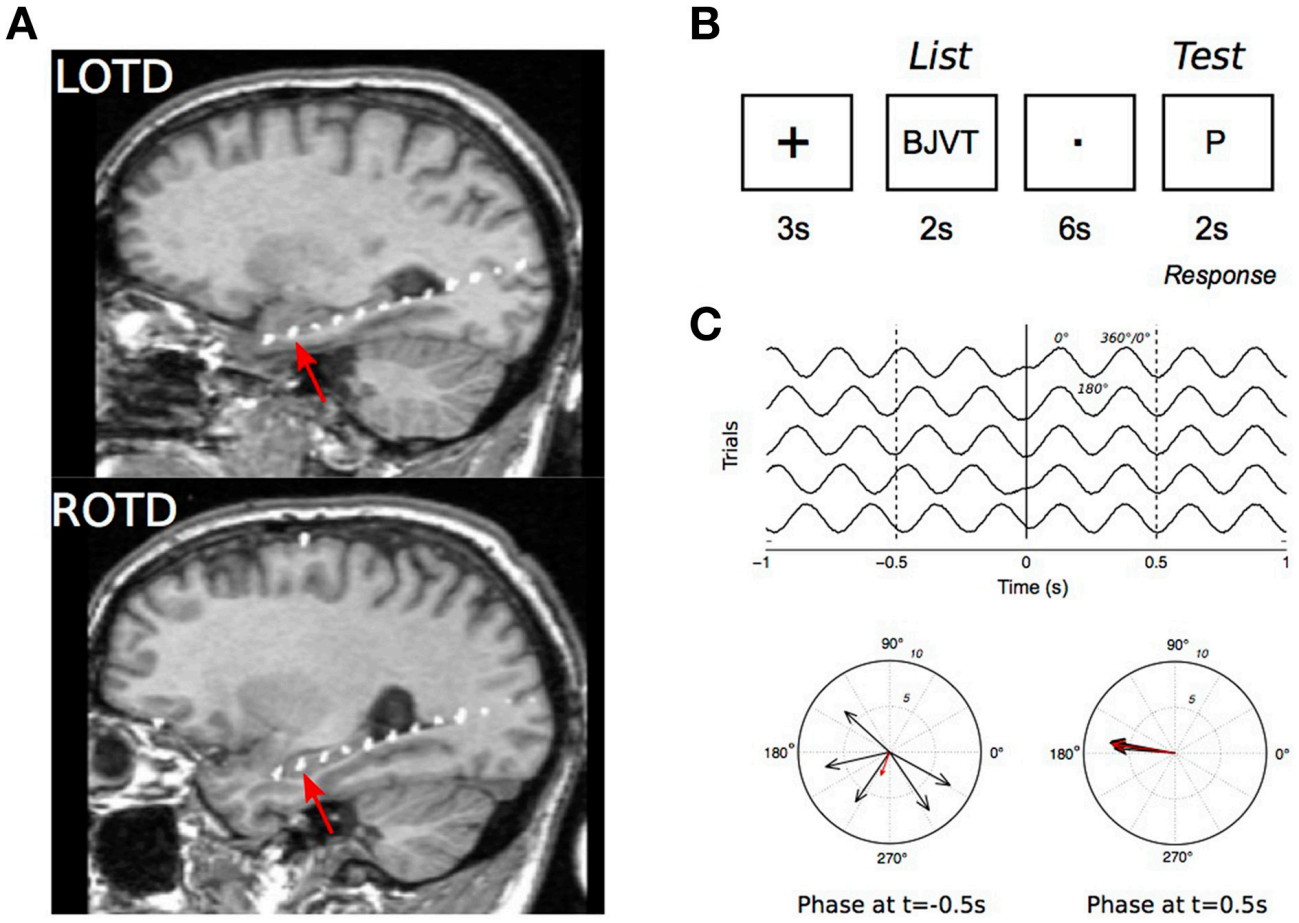
**Intracranial recordings in the Sternberg task. (A)** MRI scan of a patient showing the intracranial electrodes, with contacts in white, along the hippocampal longitudinal axes. Recordings from the electrode contact that was at the most anterior hippocampal region (red arrows) were selected for analysis. LOPD, left occipitotemporal; ROPD, right occipitotemporal depth electrode. **(B)** Stimulus presentation sequence in a trial. **(C)** Illustration of the phase reset phenomenon in simulated 4 Hz signal. Top: Simulated EEG in five trials. Signal is in phase at *t* = 0.1 s. Bottom: polar representation of EEG phase at *t* = −0.5 s and 0.5 s. Each vector represents an individual recording with vector amplitude and angle representing the signal instantaneous power and phase, respectively.

The depth electrodes were linear polyurethane shafts with platinum/iridium contacts spaced 1 cm apart (Ad-tech, Racine, WI). They were inserted via a posterior stereotaxic approach extending from the occipital cortex anteriorly through the longitudinal axis of the hippocampus. Electrode placement was confirmed with co-registration of postoperative computed tomography (CT) to preoperative magnetic resonance imaging (MRI). For each patient, we selected one electrode per implanted hemisphere located in the most anterior portion of the hippocampus. This was determined by the post-implantation MRI which shows amygdala and hippocampus. The electrode was typically the second from the distal end of the electrode chain (Figure 1A).

### Sternberg task

The Sternberg task (Figure 1B) was used to test working memory during EEG recordings (Sternberg, 1966; Kleen et al., 2013). For each trial a group of four consonant letters was displayed for 2 s on a computer screen facing the patient (“List”). A delay of 6 s followed, during which the patient fixated on a dot in the middle of the screen. A single consonant letter was then shown on the screen for 2 s (“Test”). The patients were instructed to respond with a left mouse-click (“Yes”), if they believed the “Test” letter was in the previously displayed group, and with a right mouse-click (“No”), if it was not. Trials were separated by 3 s intervals during which a “+” sign was displayed. To minimize anticipatory responses, time intervals between trials and between “List” and “Test” stage were modulated with a random jitter with a maximum variation of ±200 ms. Patients were presented with up to 120 trials a day with breaks between every 30 trials.

In order to assess the role of phase reset in memory function, we varied the memory load. For this purpose, 20% of trials were “low memory” trials in which working memory was minimal. In such trials, we used “AAAA” as the “List” and the “Test” was always a single letter “A”. Patients were asked to answer “yes” (left click) after the “Test” letter, with no discrete memory requirement aside from keeping track of the trial type.

### Data acquisition and analysis

EEG signals from the intracranial electrodes were amplified, digitized, and recorded by the clinical EEG monitoring system (Grass Technologies, Warwick, RI). The data used in this study were derived from a referential montage of electrodes, where one of the intracranial electrodes is selected as the reference to reduce motion artifacts and signal artifacts from extracranial sources. The reference electrode consisted of an additional strip electrode placed intracranially and facing the skull surface instead of the dura. Results derived from a bipolar montage and an average referential montage were also examined but were not further considered since they had similar or worse signal to noise ratio for standard power spectral analysis. Due to an upgrade of the EEG equipment during the project, the raw data were recorded with different sampling frequencies. Early patients were recorded with a maximum of 200 Hz sampling frequency, later ones up to 1600 Hz. For the early patients the cut-off frequency of the analog filter was 70 Hz, 500 Hz for the recent ones. EEG from the recently recorded patients was therefore down-sampled to 200 Hz, and the EEG analysis was restricted to a 0.5–70 Hz frequency range. The Sternberg task was synchronized with the EEG recording with Transistor–Transistor–Logic pulses on unused channels, triggered by the “List” and “Test” stimuli and patient mouse clicks (“Response”).

Trials containing high power artifacts, trials with interictal epileptiform discharges (spikes and sharp waves) and recording artifacts, were discarded from the analysis. To this end, the signal of each trial and its time derivative were ranked according to the maximum–minimum range of signal values. In most instances this allowed the removal of contaminated trials by setting a threshold between outliers (typically about 10% of the total number of trials) and the remainder of closely clustered trials. This process was aided by a custom graphical user interface implemented in Matlab allowing visual inspection of the entire set of trials for each patient and channel, to discard additional trials containing obvious and potentially disruptive signal components. The individual using the graphical user interface was blinded to other aspects of the trials (performance, phase data, etc.) to prevent biased exclusion. The percentage of rejected trials in each hemisphere is presented for each patient on Table 1.

**Table 1:** **Patient demographics**.

**Patient**	**Focus side**	**Lang. D**.	**FSIQ**	**VIQ**	**PIQ**	**% Correct (%)**	**Implant**	**Trials**	**% Artifact trials L**	**% Artifact trials R**
1	L	L > R	126	113	140	90.8	Bilateral	480	23.3	6.3
2	L	L	98	98	97	76.0	Bilateral	120	22.5	46.7
3	R	L	76	80	76	90.2	Bilateral	120	30	47.5
4	L	R > L	75	78	77	95.8	Bilateral	240	33.8	21.7
5	R	L	NA	NA	NA	94.6	Bilateral	120	24.2	24.2
6	R	L	74	72	73	97.9	Right	120	__	30.0
7	L	Bilateral	69	68	66	68.5	Bilateral	120	75.8	13.3
8	L	Bilateral	89	81	79	98.5	L	480	34.4	__
9	L	L	71	72	79	67.4	Bilateral	120	53.3	56.7
10	L > R	L > R	67	76	69	78.2	Bilateral	120	50.8	31.7

*Focus side, hemispheric location of the epileptic focus, as identified by intracranial monitoring; Lang. D, language dominance; FSIQ, Full scale intellectual quotient (IQ); VIQ, verbal IQ; PIQ, performance IQ; % correct, percentage of correct trials in the Sternberg task; L, left hemisphere; R, right Hemisphere; %Artifact Trials, percentage of trials with artifacts*.

Time-frequency representations were generated using 5-cycle Gabor wavelet transforms (Krieg et al., 2011).

To extract phase information of EEG traces, the signal was first filtered (zero-phase forward-backward Chebychev filter) in the frequency band of interest i.e., delta (0.5–4 Hz), theta (4–8 Hz), and alpha (8–12 Hz). The phase of the filtered EEG was computed using the Hilbert transform for “List” or “Test” trials across multiple trials recorded in the same electrode. For time-frequency representations (**Figures 3**, **4**), phase was estimated as the angle of the complex wavelet coefficients. Phase reset quality (i.e., how consistent the phase, at each time point was over trials) was then quantified in each condition (high and low memory, List and Test, Right and Left) using the Pairwise Phase Consistency index (PPCI) introduced by Vinck et al. (2010). We did not use the PLV used by Rizzuto et al. (2003) since it is dependent on the number of trials and overestimates population statistics for low sample sizes. In our experiment context, where conditions have different number of trials (e.g., correct vs. incorrect), this PLV could therefore bias our results. Bootstrap methods computing PLV on fixed number of trials also has disadvantages, particularly when the pool of trials is low (Vinck et al., 2010). PPCI, an alternative estimate of phase consistency, consists of determining, at all-time points during the task of determining the phase difference in all possible pairs of trials (Vinck et al., 2010).

PPC is given by:

$$\begin{array}{l} PPC_{\left(\text{t}\right)}=\frac{2}{N\left(N-1\right)}\sum_{j=1}^{N}\sum_{k=j+1}^{N}d\left(\theta j\left(\text{t}\right),\theta k\left(\text{t}\right)\right) \end{array}$$

where *d*(θ_*j*_,θ_*k*_) is the absolute angular distance between two trials defined as the function, *d*(θ_*j*_,θ_*k*_) = |θ_*j*_ – θ_*k*_|mod π. *N* is the number of trials and *t* a given time point.

We then normalized the PPC index (PPCI) as:

$$\begin{array}{l} PPCI=\left(\pi -2PPC\right)∕\pi . \end{array}$$

PPCI therefore varies between 0 and 1.

To determine whether a significant phase-locking event was observed on a given electrode, we expressed PPCIs as a baseline-referred Z-score in the following way:

$$\begin{array}{l} {Z\;PPCI}_{\left(t\right)}=\frac{{PPCI}_{\left(t\right)}-\bar{{PPCI}_{PRE}}}{{\sigma PPCI}_{PRE}} \end{array}$$

Where $\bar{{PPCI}_{PRE}}$ and σ*PPCI*_*PRE*_ are the mean and standard deviation of PPCIs computed between −1 to −0.1 s before stimulus onset. When *z* reached a value beyond 3 (*p* < 0.01), phase locking was considered significant.

For illustration purposes we also expressed phase locking using the PLV found in previous studies (Lachaux et al., 1999; Rizzuto et al., 2003, 2006). In this case, PLV is the mean resultant length of instantaneous phases across trials (Fisher, 1993).

$$\begin{array}{l} {PLV}_{t}=\frac{\sqrt{C_{t}^{2}+S_{t}^{2}}}{N} \end{array}$$

Where $C_{t}=\overset{N}{\underset{1}{\sum }}\text{cos}\left(\theta_{t}\right)$ and $S_{t}=\overset{N}{\underset{1}{\sum }}\sin\left(\theta_{t}\right)$.

*N* is the number of trials and *t* a given time point. θ is the phase of the EEG signal, here extracted from the Hillbert transform of the EEG filtered in the frequency band of interest (zero-phase forward–backward Chebychev filter).

In previous studies, a significant phase locking event was defined as the *p*-value of the Rayleigh test remaining below the critical threshold (*p* < 0.01) for at least two cycles of the respective frequency (Rizzuto et al., 2003). For the assessment of phase-reset at delta frequencies this criterion is impractical since the time resolution is reduced to intervals ranging from 0.5 to 2 s, which covers the stimulus presentations. In this study we identified phase-reset for delta frequencies if the Z-PPCI remained above 3 for more than 400 ms, which corresponds to one cycle of 2.5 Hz, i.e., the middle of the band. For θ and α, ZPPCI had to be above 3 for at least 167 and 100 ms, respectively.

Event related potentials (ERP) on correct trials were performed by averaging EEG traces for each condition (“List” and “Test”) and hemisphere (Left and Right). Resulting traces were then normalized as a *z*-score (Z-ERP) using the same equation as for Z-PPCI. Two ERP peaks were considered for analysis: a positive peak occurring around 400 ms (between 200 and 600 ms) followed by a negative peak occurring around 500 ms (between 400 and 1.1 s after stimulus presentation). These peaks are referred to as the P300 and LNC. The peak *z*-scores and times were correlated with reaction time and accuracy.

To compare PPCIs and Z-ERPs between hemispheres, stimulus type (“List” vs. “Test” and/or “High” vs. “Low” Memory Load Trials” or frequency bands we used the Generalized Estimating Equation (GEE) method. GEE is a multivariable regression method taking into account repeated measures within an individual (for most patients, two electrodes, one per hemisphere) and within electrodes (the effect of “List” and “Test” stimuli were tested on the same electrode). In addition, GEE allows potential confounding variables to be tested and the correct distribution for the data to be assumed. It is therefore less restrictive than a repeated measures ANOVA which needs an exact balance in the data and assumes a normal distribution. Data distributions were visually assessed and found to be gamma distributions in all PPCI cases and normal distributions for peak PPCI times and reaction times. We used the corresponding distributions for each outcome measure in our regression models. Goodness-of-fit was determined using the corrected quasi-likelihood under independence model criterion (QICC) and by the visual assessment of residuals. In the results section, the relationships between potential predictors and PPCIs are documented using the mean value extracted from the model, given the predictor considered. All statistical analyses were performed using SPSS (Chicago, IL).

While we alternatively use the terms “phase locking” and “phase reset,” the implications for each term are different. The term “phase locking” refers to significant increases in phase consistency of the EEG across trials. “Phase reset” rather refers to the interpretation of the phase locking, i.e., that an ongoing oscillation undergoes a systematic resetting. For instance, the systematic trigger of an evoked potential by task stimuli could induce phase consistency increases, i.e., phase locking, without phase reset.

## Results

Table 1 shows the demographic and clinical characteristics of the 10 patients who participated in the study. Each participant performed an average of 188.2 ± 45.8 trials, and had an average of 10.04 ± 2.6% errors.

### Characteristics of the EEG signal

**Figure 2:**
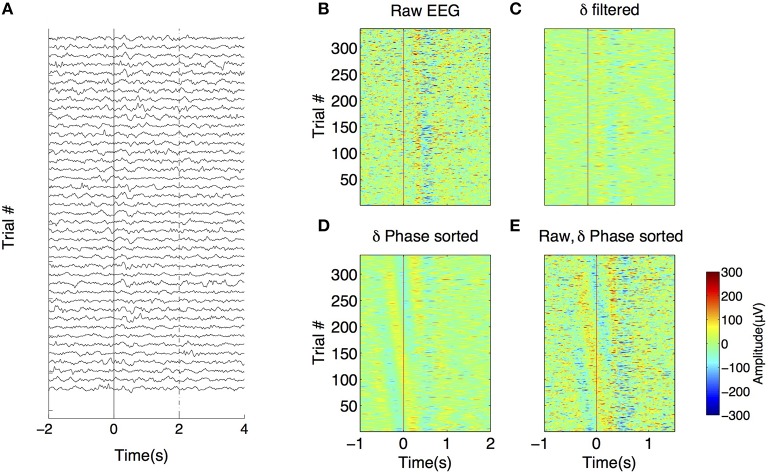
**Phase reset in intrahippocampal recordings. (A)** EEG recorded in 40 trials from the right hippocampal electrode of patient 1. X-axis corresponds to the time relative to the presentation of the “Test” stimulus (time 0, red line). **(B)** False color graph of EEGs (filtered 0.1–100 Hz) from the same electrode during test presentation for the whole session. Color axis represents EEG amplitude. Note the systematic presence of a peak followed by a trough at ~200 ms. **(C)** Same data filtered in delta (0.1–4 Hz), **(D)** Same as in panel **(D)** but trials are sorted by increasing phase 0.1 s before stimulus onset. **(E)** Unfiltered recordings with trials sorted by increasing delta phase 0.1 s before stimulus onset.

Figure 2A shows EEG traces (filtered between 0.1 and 70 Hz) from the same electrode (right electrode in Patient #1) taken from 40 trials at the time of stimulus (“List”) presentation. EEGs from all trials in the same electrode are viewed on a false color plot (Figure 2B), with EEG amplitude on the color axis, time in abscissa and trial number in ordinates. There is an alignment of negative peaks (in blue) at 0.5 to 0.6 s after stimulus onset. Figure 2C shows similar representations but with filtered signals (same electrode and stimulus presentation) in delta. Phase locking of delta oscillations is demonstrated in Figure 2D where unfiltered trials are sorted according to the phase of delta 100 ms before stimulus onset. This caused the negative and positive peaks (in blue and yellow, respectively) to be organized in a diagonal band. Starting at ~200 ms, these bands are disrupted and a negative, vertical band appears.

**Figure 3:**
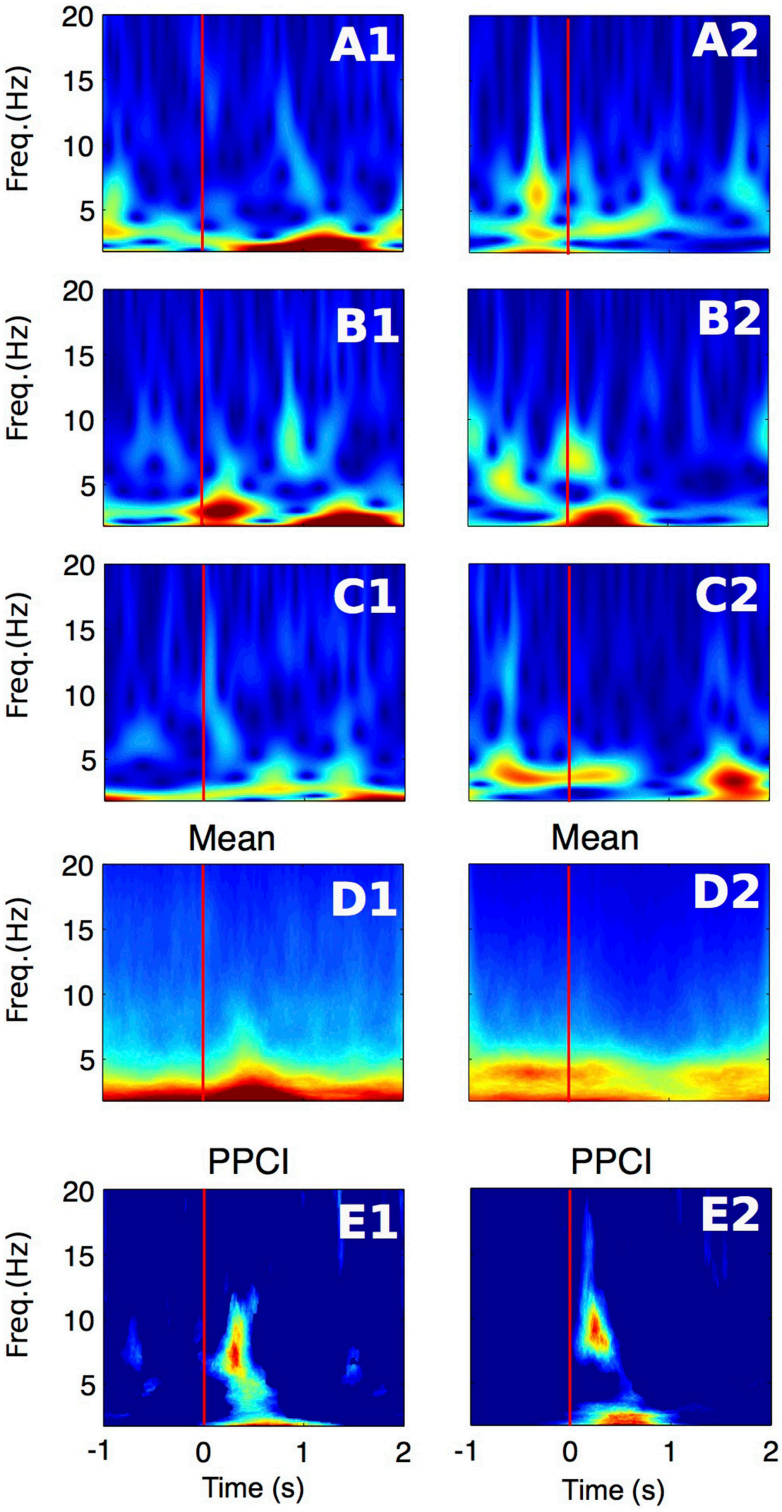
**Time-frequency representations of the EEG signal recorded in the right hippocampal electrode of two patients (1–2). (A–C)** Wavelet spectrogram of three successive trials in two patients (1–2). Color scale (from blue to dark red) for panels **(A1–D1)**: 0–70 μV^2^/Hz, for panels **(A2–D2)**: 0–30 μV^2^/Hz **(D)** Average power for the whole session in the same electrodes. Color scale same as in panels **(A–C). (E)** Time-frequency representation of PPCI in all bands. Here, phase was extracted directly from the wavelet coefficients computed in all trials. Color scale (from blue to dark red) for panel **(E1)**: 0–0.09, for panel **(E2)**: 0–0.14.

Examples of spectrograms (computed with the Gabor wavelet transform) of EEGs taken in three successive trials are shown in Figures 3A–C for Patient #1 (Figures 3A1–C1) and patient #8 (Figures 3A2–C2). The whole session power average is shown in Figures 3D1,D2. As can be seen in these two extreme cases, the evolution of power over time is variable between patients and from trial to trial. For instance, in patient 8, delta power decreases ~500–1 s after stimulus onset (List here). For Patient #1, average delta power increases after stimulus onset (from 0 to 1 s). PPCIs computed from the phase of the wavelet transform across frequencies are shown in Figures 3E1,E2.

**Figure 4:**
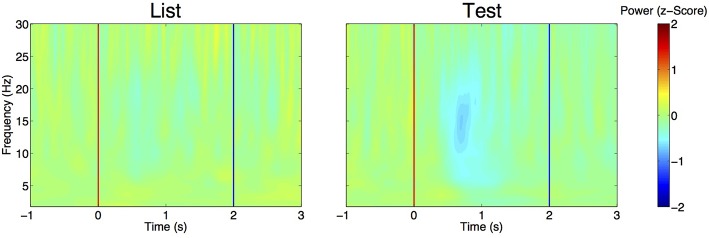
**Evolution of EEG power over time aligned to List (left) and Test (right) stimulus onset (red line)**. For each frequency band, power is expressed as a z-score, with means and standard deviations estimated from pre-stimulus baseline (–1 to 0 s) across trials. *Z*-scores are then averaged across trials, electrodes and patients. Note the decrease in z-score for frequencies ranging from 5 to 20 Hz at *t* = 0.5 to 1 s. Blue line: time at which stimulus is removed from the screen.

We first compared the EEG power (computed with wavelet spectrograms) in delta, theta and alpha between the period before (−2 to −1 s) and after (0.5–1 s) after stimulus presentation. Using GEE with repeated measures and stimulus type (List vs. Test), laterality (Right or Left hemisphere) and period (before vs. after) as covariates, we found that, in all frequency bands, power was decreased after stimulus presentation as compared to before (Table 2). In all bands, there was a significant interaction between stimulus type and period analyzed, i.e., power decrease was more pronounced after Test presentation than after List. This effect is demonstrated in Figure 4 that shows time frequency plots of the *z*-scores of EEG power. Here, *z*-scores for each frequency point and each time points are computed on the basis of the mean and standard deviation extracted from pre-stimulus baseline (−1 to 0 s). *Z*-scores are then averaged across trials, hemispheres and patients. Figure 4 therefore represents the time evolution of EEG power, in each frequency for List and Test stimuli. As in Rizzuto et al. (2003, 2006), there is a noticeable decrease in power, occurring between 0.5 and 1 s after Test stimulus presentation in frequencies ranging from 5 to 20 Hz.

**Table 2:** **Average power in μV^2^/Hz, in theta, delta, and alpha for before (Pre-Stim: −2 to −1 s) and after (Post-Stim: +0.5 to +1 s) stimulus presentation**.

	**Overall**		**List**		**Test**	
	**Pre-Stim**	**Post-Stim**	**Pre-Stim**	**Post-Stim**	**Pre-Stim**	**Post-Stim**
Delta	34.34 ± 4.01	32.55 ± 3.79^*^	33.43 ± 4.01	33.15 ± 4.01	37.38 ± 4.55	31.96 ± 3.69^†^
Theta	21.54 ± 3.24	18.67 ± 2.81^***^	20.31 ± 2.9	19.81 ± 3.03	22.86 ± 3.55	17.58 ± 2.66^†^
Alpha	13.47 ± 1.8	11.49 ± 1.65^***^	12.81 ± 1.67	11.84 ± 1.56	14.18 ± 2.01	11.15 ± 1.77^†^

Significance levels presentation (GEE) for comparisons between Pre and Post stimulus are indicated in the following way:

* *p < 0.05*,

*** *p < 0.001*;

*Stimulus type x Period interaction*:

† *p < 0.01*.

### Phase locking

We then analyzed phase locking for each patient, with EEG filtered in delta, theta and alpha frequency bands. Phase was estimated for each time bin ranging from 0 to 1.1 s after the “List” and “Test” stimulus presentation.

**Figure 5:**
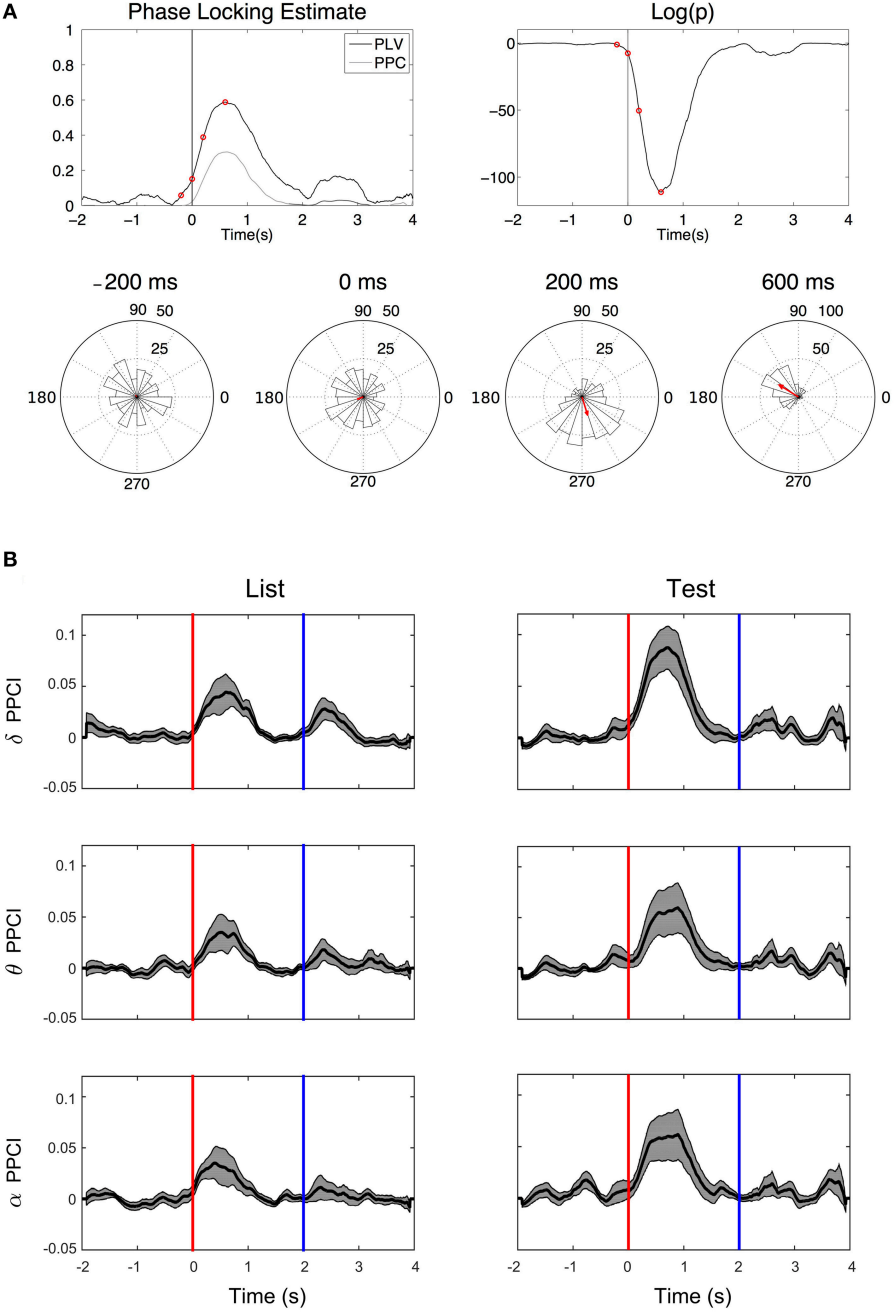
**Phase reset. (A)** Top left: Time progression of the PPCI and phase locking value (PLV derived from Rayleigh statistics). Top right: log of the significance *p*-value (top right) of hippocampal EEG filtered in the delta range amongst trials for a single patient from a single electrode. Time 0 corresponds to stimulus presentation (“Test”). Bottom circular histograms show phase histograms at different times around stimulus presentation (red circles on the PPCI plots). Red arrows correspond to the mean phase vectors. Here, phase 0 corresponds to the peak of the oscillation (3 o'clock on the trigonometric circle by convention). **(B)** Progression of mean PPCI (±sem) averaged across patients and electrodes in delta (δ), theta (θ), and alpha (α) relative to “List” (left) and “Test” (right) stimulus presentation. Red line: time of stimulus onset. Note also the presence of phase reset at the time when the stimulus is removed from the screen (Blue line).

Figure 5A illustrates the time progression of phase consistency across trials for delta filtered EEGs from one patient. Both PPCI and phase locking value (PLV derived from the Rayleigh statistics) are represented along with the corresponding to significance levels over time (*p*-value). Phase histograms show the progressive buildup of phase concentration over time. Figure 5B shows the progression of the PPCI averaged across patients and electrodes as a function of stimulus presentation (“List” and “Test”) in the three bands.

When enough trials (*n* > 20) were available to compute PPCI in a given condition (two cases per hemisphere i.e., list or test), EEG recordings from the intra-hippocampal electrodes were found to show significant phase locking (peak Z-PPCI >3 for either “List” or “Test” items) in delta (6/8 cases in left; 9/9 electrodes in the right hemisphere); theta (3/8 cases in left, 8/9 cases in the right hemisphere); and alpha (5/8 cases in left, 4/9 in the right hemisphere). Here, PPCIs were expressed as a z-score from pre-stimulus baseline (see Methods Section). *T*-tests performed on all cases combined (List and test, right and left, *n* = 34), showed a significant deviation of peak Z-PPCIs from values expected by chance (*z* ≤ 3, which is expected with an α ≤ 0.001) in all bands (mean *z* in delta: 29.51 ± 9.34, *t* = 2.84, *p* ≤ 0.01; mean *z* in theta: 7.31 ± 1.37, *t* = 3.16, *p* ≤ 0.01; mean *z* in alpha: 5.93 ± 1.27, *t* = 2.29, *p* ≤ 0.01). Pooling electrodes from both hemispheres and all types of stimuli, PPCI reached its highest value 0.593 ± 0.039 ms after the stimulus presentation for delta; 0.499 ± 0.055 ms for theta and 0.441 ± 0.052 ms for alpha. Peak time for PPCI was significantly later in delta than in theta [paired *t*-test: *t*_(33)_ = 2.335; *p* = 0.026] but did not differ significantly from alpha peak time [*t*_(33)_ = 1.43; *p* = 0.16].

For all the following GEE-based analyses, we investigated whether PPCIs or PPCI peak times are significantly affected by specific factors. We refer to “Stimulus type” when considering whether there is a difference in PPCI after “List” vs. “Test” presentation and “Laterality” when considering whether PPCI differ when EEGs are recorded in the right or the left hemisphere. To compare phase locking quality between conditions and bands, we considered the peak PPCI and its timing within a time window ranging from 0 to 1.1 s after stimulus presentation. Using GEE (using multiple observations, stimulus type and laterality as covariates) we found that the peak time for the PPCI in delta and alpha was earlier in “List” than in “Test” (delta: *t* = 0.519 ± 0.047 vs. 0.661 ± 0.047 ms, *p* ≤ 0.005; alpha: *T* = 0.373 ± 0.050 vs. 0.505 ± 0.048, *p* ≤ 0.005), but did not differ significantly according to the hemisphere.

We then tested whether the peak PPCIs differed between bands, i.e., whether phases were more consistently aligned after stimulus presentation in delta as compared to theta and alpha. Using GEE (using repeated measures, stimulus type and laterality as covariates) we found that PPCI in was greater in delta (PPCI = 0.143 ± 0.014) than in theta (PPCI = 0.071 ± 0.009; paired *t*-test: *p* ≤ 0.001) and alpha (PPCI = 0.064 ± 0.006; *p* ≤ 0.001) but did not differ between theta and alpha (*p* = 0.39). To summarize, significant phase locking was observed in all three bands but was more robust in delta.

### Phase locking, stimulus type, and laterality

We then analyzed the effect of both stimulus type and laterality on peak PPCI in each of the three bands (GEE using repeated measures and both factors as covariates). In theta or in alpha, peak PPCIs did not differ between stimulus type (“List” and “Test”) nor hemisphere (Right or Left). For delta, there was a significant effect of stimulus type (estimated peak PPCI in “List” = 0.084 ± 0.0155 vs. 0.157 ± 0.0157 in “Test,” *p* = 0.002) but not of laterality (estimated peak PPCI in right = 0.097 ± 0.007 vs. 0.137 ± 0.028 in left, *p* = 0.09), and the interaction between those two factors was not significant (*p* = 0.24). In theta, PPCI was higher in the right hemisphere (estimated peak PPCI in right = 0.060 ± 0.013 vs. 0.033 ± 0.006 in left, *p* = 0.01), but there was no significant effect of stimulus type nor significant interaction. Finally, there was no effect of the two variables on PPCI in alpha. In summary, delta phase locking was stronger after presentation of “Test” stimuli and theta phase locking was stronger in the right than in the left hemisphere.

### Phase locking and laterality of the epileptic focus

Phase locking was observed at a similar rate in the electrodes located in the epileptic (the temporal lobe from which seizures began) and in non-epileptic temporal lobe. This was the case for signals filtered in delta (there was a significant phase locking in 50% of the electrodes in the epileptic vs. 59% in the non-epileptic side, respectively, *X*^2^ = 0.33, NS), theta (significant phase locking in 31% of the electrodes in the epileptic vs. 32% in the non-epileptic side, *X*^2^ = 0.01, NS) and alpha (significant phase locking in 18% of the electrodes in the epileptic vs. 32% in the non-epileptic side, *X*^2^ = 0.08, NS). There was no difference in peak PPCI (within a 0–1 s window post-stimulus) between the hippocampus ipsilateral to the focus vs. the contralateral one in neither of the bands (GEE using repeated measures and stimulus type and epileptic vs. non-epileptic side as covariates); mean delta peak PPCI in the epileptic side: 0.113 ± 0.0130 vs. 0.123 ± 0.031 in the contralateral side (*p* = 0.76), mean theta peak PPCI in the epileptic side: 0.047 ± 0.012 vs. 0.050 ± 0.008 in the non-epileptic side (*p* = 0.82; mean alpha peak PPCI in epileptic side: 0.056 ± 0.009 vs. 0.046 ± 0.002 in the non-epileptic side (*p* = 0.21).

### Memory and phase locking

**Figure 6:**
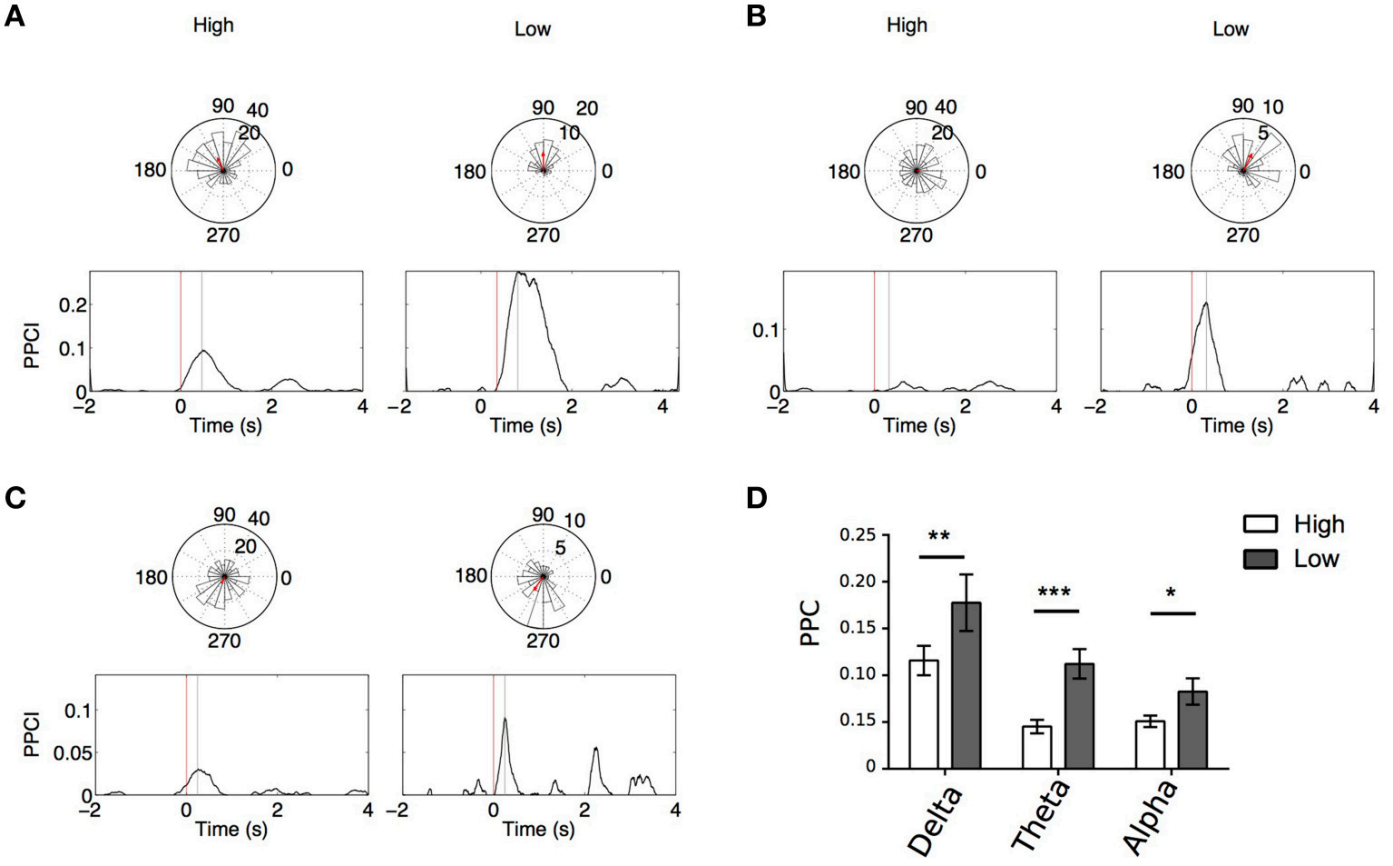
**(A–C)** Example, for the same electrode, of PPCI progression over time in “High” vs. “Low” memory trials in delta for List trials in one patient **(A)**; theta for List trials in one patient **(B)**; alpha for List trials in one patient **(C)**. Top: Phase histograms taken at the time where PPCI reached its peak (gray line). **(D)** Mean peak PPCI across patients between correct “Test” and “Lure” trials in the three bands. ^*^p < 0.05; ^**^p < 0.01; ^***^p < 0.001.

Figures 6A–C illustrates PPCI examples of the same electrode between “High Memory Load Correct Trials” and “Low Memory Correct Trials.” In a majority of these cases, significant phase locking was also observed in low memory trials. Using GEE (with repeated measures and Trial type, stimulus type, and laterality as covariates) we compared PPCI in “High Memory Load Correct Trials” and “Low Memory Correct Trials.” Because fewer of the low memory trials were presented to patients and because interictal abnormalities decreased the number of trials to < 20 in some patients (our minimum trial threshold for PPCI analysis), we only were able to analyze data from six patients. Here, we combined peak PPCIs obtained in “List” and “Test” presentations and Left and Right hemisphere when patients had bilateral implants. This led to either four (unilateral implants) or eight measures (bilateral implants) per subject. We found that phase locking was significantly higher in “Low Memory Correct Trials” than in “High Memory Correct Trials” in all bands (Delta: mean peak PPCI in “High Memory Correct Trials” = 0.112 ± 0.013 vs. 0.177 ± 0.029 in “Low Memory Correct Trials,” *p* = 0.003; Theta: mean peak PPCI in “High Memory Correct Trials” = 0.045 ± 0.007 vs. 0.118 ± 0.019 in low memory trials, *p* < 0.0001; Alpha: mean peak PPCI in “High Memory Correct Trials” high = 0.051 ± 0.006 vs. 0.082 ± 0.014 in “Low Memory Correct Trials,” *p* = 0.009) (Figure 6D). When considering “Low Memory Correct Trials” (using GEE with repeated measures and stimulus type and laterality as covariates), there was no effect of laterality or stimulus type in delta or theta. However, in alpha, “Low Memory Correct Trials” PPCI was higher in “List” presentation (alpha: mean peak PPCI in “List” = 0.088 ± 0.0123 vs. 0.069 ± 0.014 in “Test,” *p* < 0.048). No effect of laterality was observed. As expected from their low memory load, reaction time was significantly shorter in “Low Memory Correct Trials” than in “High Memory Correct Trials” [*RT* = 1.45 ± 0.13; “Low Memory Correct Trials” = 0.81 ± 0.14; paired *t*-test, *t*_(10)_ = 7.59, *p* < 0.0001].

### Performance and phase locking

The number of trials with incorrect answers (Error trials) was very low, and further limited by exclusion of those with interictal discharges. This limited the statistical analysis related to performance as only two patients had enough error trials to perform the analysis. It is therefore not possible to draw conclusions from such a low number of participants. Of note, one patient showed robust phase locking in all bands, hemisphere, and stimulus type in correct trials. In error trials however, PPCI during “List” failed to increase and reach significance, whereas, during “Test” there was robust phase locking.

**Figure 7:**
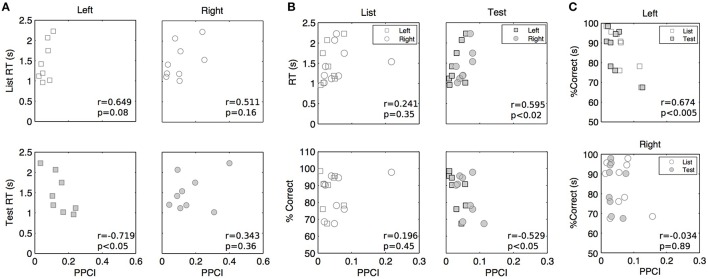
**Peak post stimulus PPCI vs. performance in delta (A), theta (B), and alpha (C)**. Each point represents peak PPCI measures for both hemispheres and stimulus type vs. performance of a given patient. Performance is computed on the basis of artifact free trials. Pearson correlation coefficients (*r*) and associated *p*-values are shown for illustrative purposes.

Mean peak PPCIs (within a 0–1 s window post stimulus) did not differ between hits and correct rejections in delta (Test presentation only, GEE using repeated measures with laterality and hit/rejections as covariates, mean peak PPCI for Hits: 0.151 ± 0.021 vs. 0.198 ± 0.014 for rejections; *p* = 0.859), theta (mean peak PPCI for Hits: 0.074 ± 0.008 vs. 0.064 ± 0.008 for rejections; *p* = 0.27), or alpha (mean peak PPCI for Hits: 0.075 ± 0.012 vs. 0.053 ± 0.008 for, rejections; *p* = 0.095). To further investigate the functional relevance of phase locking, we asked whether reaction time (RT) or accuracy (percent correct) varied with peak PPCI-values (within a 0–1 s post stimulus window) in delta, theta, or alpha. For each patient, hemisphere, and condition, mean RT and accuracy were computed on the basis of artifact-free trials. Several observations were made using GEE with PPCI, stimulus type and laterality as covariates: (a) There is a significant three-way interaction between the effects of delta peak PPCI, stimulus type and laterality on reaction time (*p* < 0.004). This is characterized by a negative relationship between delta peak PPCI and RT in the left hemisphere only during test trials (Figure 7A); (b) RT is significantly correlated to theta peak PPCI (*p* = 0.001) and this is characterized by a positive relationship during “Test” trials but not during “List” trials (difference in slopes *p* < 0.001, Figure 6, top); (c) There is no significant relationship between RT and alpha PPCI; (d) there is no significant relationship between accuracy and delta PPCI; (e) There is a significant correlation between theta PPCI and accuracy (*p* = 0.017), higher theta PPCI predicting lower accuracy (Figure 7B). This effect is only observed during “Test” stimulus presentation (*p* = 0.003); (f) There is a significant invert relationship between alpha PPCI and accuracy on recordings taken from the left hemisphere (Figure 7C; *p* = 0.012).

### The eventual involvement of an ERP and its relationship with performance

The fact that phase locking occurs simultaneously in multiple, neighboring bands (Figure 3E) suggests that instead of a genuine phase reset of ongoing oscillation, the observed phenomenon is in fact caused by a systematic single evoked potential appearing at a fixed time after stimulus onset. In addition, there are instances where increases of PPCIs are associated with increased power of oscillations (Figure 3D). Therefore, it is possible that a single event, appearing at a fixed time after stimulus onset, is mistaken for an oscillatory pattern (both after filtering or wavelet transform) and leads to an increase in PPCI. We therefore considered this eventuality and performed traditional ERP analysis on intra-hippocampal recordings.

**Figure 8:**
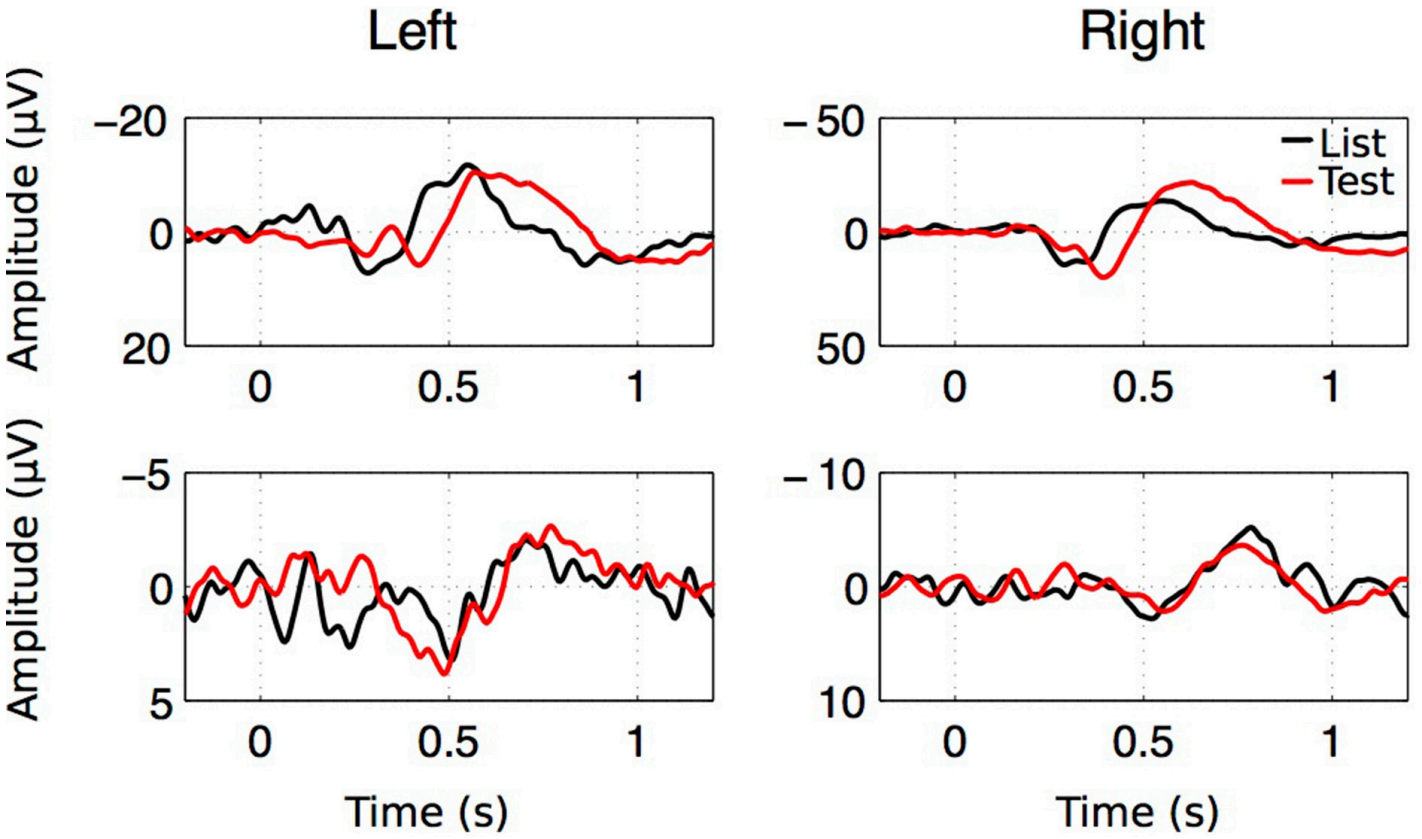
**ERP examples for both hemispheres, both stimulus types in two patients**. Despite the large amplitude and latency variability of the ERP components, two successive components were observed. A first positive component with a latency going from 0.2 to 1.1 s (P300) followed by a large negative component (LNC) starting after 0.4 s.

As seen on Figure 8, averaging EEGs aligned to stimulus onset leads to strong ERPs that are strikingly similar to those published by Mormann et al. (2005). These consist of a positive component appearing within 300 ms after stimulus onset (P300), followed by a late negative component (LNC) occurring after 400 ms. Across patients, P300 reached an average z-score of 4.0 ± 0.67 at *t* = 0.399 ± 0.0206 s after stimulus onset. The LNC *z*-score reached −4.39±0.75 at *t* = 0.639 ± 0.0273 s post-stimulus.

GEE analyses revealed that LNC *z*-scores (absolute values) are significantly greater in the right (*z* = 5.18 ± 1.42) vs. the left hippocampus (*z* = 3.60 ± 0.94, *p* < 0.05) and greater after “Test” (*z* = 4.96 ± 1.29) than after “List” (*z* = 3.76 ± 0.99, *p* < 0.05). There is no significant effect of laterality or stimulus type on LNC time, P300 *z*-scores or P300 time.

We then investigated if there is a relationship between ERP parameters and performance measures (reaction time and accuracy). We performed GEE analyses using repeated measures and using laterality and stimulus. The relationships between potential predictors and performance is documented here using their slope (β) and standard error. *Z*-scores were log transformed and accuracy scores were transformed to fit a gamma distribution. There is a significant relationship between P300 *z*-scores (log transformed) and accuracy (β = 0.109 ± 0.039, *p* < 0.01) and reaction time (β = −0.223±0.0667, *p* < 0.001), LNC *z*-scores and accuracy (β = 0.067 ± 0.028, *p* < 0.05) and reaction time (β = −0.175±0.059, *p* < 0.01).

ERP amplitude of both P300 and LNC were significantly decreased in low memory trials as compared to high memory trials (P300 went from *z* = 3.89 ± 0.84 in high to *z* = 2.63 ± 0.60 in low memory trials, *p* < 0.001; and LNC went from *z* = 4.26 ± 1.12 in high to *z* = 2.62 ± 0.53 in low memory trials, *p* < 0.001). However, ERP analyses rely on the number of trials to increase signal to noise ratio. Indeed, noise in an average ERP decreases as a function of the square root of the number of trials (Luck, 2005). Therefore, given the “noise” amplitude in single trials (45 μV in average) and the LNC amplitude (60 μV in the best cases), it is estimated that 50 trials are required in order to provide an ERP signal to noise ratio of 10 (noise in an average decreases as a function of the square root of the number of trials). As our statistics are based on *z*-scores estimated from pre-stimulus periods where only noise is present, low number of trials could result in artificially lower *z*-scores. While this requirement was reached in all instances in high memory load, it was only reached in low memory trials for two patients. We therefore interpret this decrease in *z*-scores with caution.

**Figure 9:**
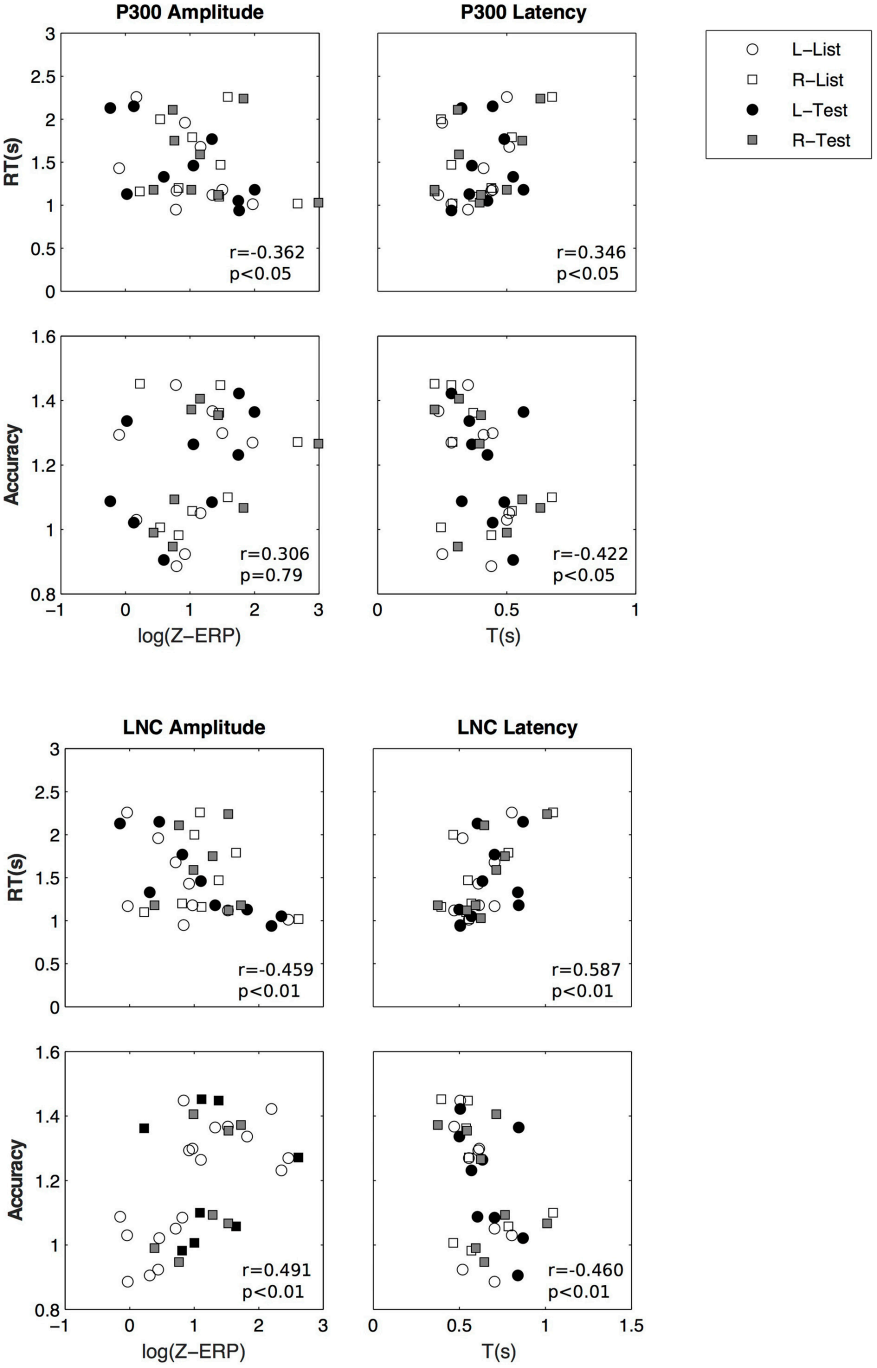
**Amplitude and latency of P300 (top) and LNC (bottom) vs. reaction time and accuracy**. Pearson correlation coefficients (*r*) and associated *p*-values are shown for illustrative purposes.

In summary two ERP components were isolated from averaged EEGs across trials. The LNC had a higher amplitude in the right hemisphere and was greater after “Test” presentation. For both LNC and P300, amplitude and time of ERPs were correlated with accuracy and reaction time (Figure 9).

## Discussion

In this study, we investigated whether hippocampal phase reset of oscillations after stimulus presentation in a working memory task was correlated with performance. In agreement with other studies (Mormann et al., 2005; Rizzuto et al., 2006) phase locking, measured by the PPCI (Vinck et al., 2010), was observed simultaneously in multiple bands, including delta, theta, and alpha frequency bands. Contrary to our expectations, PPCIs were higher in low memory than in high memory trials. Between patients however, high delta PPCIs in the left hemisphere were associated with faster reaction times. In contrast, higher theta and alpha PPCI predicted either slower reaction times or worse performance.

Altogether, our results suggest that phase reset plays a relevant role in working memory performance. However, the increased PPCI-values in low memory trials suggest that it is not specifically involved in actual processing of memory information but rather in a more general synchronizing event induced by task relevant stimuli (Mormann et al., 2005). In the rodent hippocampus, stimulus-induced phase reset of ongoing theta oscillations is a robust phenomenon that is only observed in working memory and absent in reference memory tasks (Givens, 1996). In humans, phase reset is also believed to promote inter-regional communication by synchronizing oscillations in structures participating in the same task (Lakatos et al., 2009; Mercier et al., 2013). We propose that phase reset in the Sternberg task plays a similar role, i.e., it synchronizes networks (within the hippocampus and connected structures such as the prefrontal cortex) that are involved in working memory. Higher phase reset between subjects may then represent a better ability to synchronize networks to process sensory information, explaining faster reaction times. It is also possible that phase reset is a “wiping” process by which previous items in working memory are cleared to allow new items to be processed (Gray and McNaughton, 2000).

PPCI relationship with performance suggest that delta phase locking is more relevant to the task than theta or alpha: PPCIs are greater in delta and patients with better delta PPCIs are have faster reaction times. In contrast, patients with better theta PPCIs tend to be slower in the task and make more errors. Further, delta PPCI was higher during test, suggesting a greater involvement in recall. The relevance of delta oscillations in the temporal lobe has been shown in several human studies (Bodizs et al., 2001; Jacobs et al., 2007; Watrous et al., 2011). Single unit firing in the human temporal lobe is phase locked to delta oscillations (Jacobs et al., 2007), supporting the notion that neuronal coding is coordinated in this frequency in the human hippocampus. Furthermore, human hippocampal delta, but not theta as expected from rodent physiology, is associated with REM sleep (Bodizs et al., 2001), also suggesting that delta plays a role in memory processes occurring during sleep. Indeed, there is accumulating evidence suggesting that delta oscillation in humans could well be functionally analogous to the rodent theta rhythm. In agreement with this hypothesis, our results support the notion that in humans, delta plays a role similar to rodent theta in information processing. On the other hand, that phase locking in alpha is associated with poorer performance goes in favor of a standard view that alpha oscillations constitute an “idling” rhythm (Pfurtscheller et al., 1996) or the one defended by Klimesch et al. (1999) proposing that alpha rhythms represent a state of disengagement of the structures that generate them. Therefore, higher alpha phase reset in some of our patients would be a signature of hippocampal disengagement in the task, i.e., worse performance.

Contrary to rodents where hippocampal delta and theta are mutually exclusive, oscillations in theta, delta, and alpha were present during the task performance. Co-occurrence of multiple, often coordinated frequency bands, have been documented in rodents and humans in hippocampal and cortical networks during various working or long-term memory tasks (Raghavachari et al., 2001; Rizzuto et al., 2003; Gruber et al., 2005; Fujisawa and Buzsaki, 2011; Bieri et al., 2014). It is proposed that specific frequency bands are associated with distinct information processing modes and that the coordination of multiple oscillations reflect the integration of this information as well as the communication with brain structures involved in these processes (Sauseng and Klimesch, 2008; Colgin and Moser, 2010; Benchenane et al., 2011). Similarly, our results, like previous studies, show that phase reset can occur at multiple frequencies simultaneously in the temporal lobe (Rizzuto et al., 2003, 2006). Phase reset in different frequencies may reflect the synchronization of oscillations involving distinct information processes across several anatomical structures.

While PPCI and other phase locking indices used in previous studies (Rizzuto et al., 2003, 2006; Mormann et al., 2005) appropriately estimate phase concentration at a given time after stimulus presentation, they cannot ensure that the observed phase locking is caused by an actual reset of ongoing oscillations or by an evoked potential. As for phase reset, intrahippocampal ERPs have been studied in the context of working memory tasks (Mormann et al., 2005; Axmacher et al., 2007, 2010). Late ERP components have been shown to significantly vary with cognitive demand. For instance, Mormann et al. (2005) showed that the intrahippocampal LNC, observed 400 ms post-stimulus, was higher after hits than after correct rejections in a word recognition task. Similarly, Axmacher et al. (2007, 2010) found that slow (500–1200 ms) DC negative potentials were significantly more negative in high than in low memory load conditions in a Sternberg task. Although our filtering protocol did not allow us to study the latter type of DC potentials, we found ERP patterns identical to those described by Mormann et al. (2005). Across patients, higher ERP components were correlated with faster reaction times and better working memory performance.

The performance relevance of both phase consistency indexes and ERP components in our task lead us to consider two opposite models of ERPs in electroencephalography. On the one hand, the classical model (Luck, 2005) considers ERPs as an additive waveform on top of background noise or unsynchronized oscillations. On the other hand, the phase hypothesis states that the evoked activity arising from the ERP analysis is generated by a time-locked phase resetting of one or several ongoing oscillations (Klimesch et al., 2004, 2007; Sauseng et al., 2007). In our case, the P300-LNP complex could also be generated by successive locking of theta and delta oscillations (Mormann et al., 2005). Distinguishing between the two hypotheses is complex, particularly when oscillations in multiple, neighboring bands are present and when variations of power occur at the time of high phase locking/ERP. While several methods have been proposed to test these hypothesis in experimental data (Sauseng et al., 2007; Krieg et al., 2011), none of these methods allowed us clearly state that an ERP or a phase reset was present in the absence of the other (data not shown). However, at the time when phase locking reaches its maximum there is, across trials and patients, a transient drop in frequency power in all bands (see Figure 4 and Results Section). This observation goes against the ERP hypothesis and in favor of a phase reset for two reasons. First, the systematic occurrence of an evoked potential would induce a transient power increase, not decrease for the frequency bands that show significant phase locking. As in Rizzuto et al. (2003, 2006), this was not observed and therefore contradicts this hypothesis. Second, the transient decrease in power that we observed is expected by the phase reset scenario. Indeed, phase reset, by definition consists of a transient disruption of the ongoing oscillation in order to force it to be aligned to a different phase. Such disruption would therefore cause a transient decrease in power, similar to the one observed in Figure 4.

That said, we cannot completely rule out the possibility that an evoked potential is present in our data. It is actually plausible to think that both phase reset of ongoing oscillations and ERPs coexist. Indeed, such scenario has been previously reported in a study by Givens (1996) who trained rats to either perform a working memory or a reference memory task using the same experimental stimuli in both tasks. Rat intra-hippocampal recordings showed not only that theta phase reset was only observed in the working memory task but that theta was strongly phase locked for five or more cycles after stimulus onset. Importantly, the average evoked response in reference memory trials revealed an initial evoked potential and the absence of resetting of theta activity. The same evoked potential was also observed in working memory trials but was mixed with theta activity. Therefore, evoked potentials and phase reset can occur either independently or simultaneously. Given that our task is a working memory task, a similar scenario is likely to be at play.

In conclusion, our results suggest that phase reset in delta, rather than theta plays a role in performance in the Sternberg task and that this role is more related to readiness of the network than in a direct working memory processing. We propose that phase reset in different frequency bands reflects synchronization of different but overlapping networks within the hippocampus and connected structures sub-serving different functions. We do acknowledge, however the possibility that the observed phase locking events are caused, even partially by the presence of a systematic potential evoked by the signal. In this scenario, the components of this potential may play a similar role that the one we attribute to phase reset.

## Author contributions

PL, JK, and BJ contributed to experimental design, data acquisition, data analysis, and manuscript preparation. MT and RS contributed to data analysis and manuscript preparation. GH contributed to experimental design and manuscript preparation. DR contributed to data acquisition and manuscript preparation.

### Conflict of interest statement

The authors declare that the research was conducted in the absence of any commercial or financial relationships that could be construed as a potential conflict of interest.
